# Synthesis and crystal structure of 2-amino-4-(3-isobutyl-1-phenyl-1*H*-pyrazol-4-yl)-7,7-dimethyl-5-oxo-5,6,7,8-tetra­hydro-4*H*-chromene-3-carbo­nitrile

**DOI:** 10.1107/S2056989026006973

**Published:** 2026-07-16

**Authors:** Hagar T. Salama, Mamdouh A. Abu-Zaied, Rasha A. Azzam, Ali M. S. Hebishy, Galal H. Elgemeie, Peter G. Jones

**Affiliations:** a Chemistry Department, Faculty of Science, Capital University, Helwan, Egypt; b Green Chemistry Department, National Research Centre, Dokki, Giza, Egypt; c Institut für Anorganische und Analytische Chemie, Technische Universität Braunschweig, Hagenring 30, D-38106 Braunschweig, Germany; Universität Greifswald, Germany

**Keywords:** crystal structure, chromene, pyrazole, hy­dro­gen bonds, twinning

## Abstract

The title com­pound, C_25_H_28_N_4_O_2_, crystallizes in space group *P*1 with *Z* = 4. The two independent mol­ecules are related by the approximate translation (*x* + 1, *y* + 1, *z*), but differ in the orientation of the phenyl substituent. Crystals are non-merohedrally twinned by a 180° rotation about c*. In the modified chromene ring systems, the two formal double bonds are the shortest C—C bonds. The oxo ring displays a flattened boat conformation, whereas the other ring is approximately a flattened envelope, with the CMe_2_ C atom out of the plane. The mol­ecules are connected by classical N—H⋯N_pyrazole_ hy­dro­gen bonds to form a ribbon of alternating independent mol­ecules parallel to [110]. The ribbons are joined by weak C_pyrazole_—H⋯N_nitrile_ hy­dro­gen bonds to form a layer structure parallel to the *ab* plane in the region *z* ≃ 3/4.

## Abstract 2

The two independent mol­ecules of the title chromenecarbo­nitrile are related by approximate translation but differ in the orientation of the phenyl substituent. They are connected by classical N—H⋯N_pyrazole_ hy­dro­gen bonds to form a ribbon. The ribbons are joined by ‘weak’ C_pyrazole_—H⋯N_nitrile_ hy­dro­gen bonds to form a layer structure.

## 1. Chemical context

Chromene derivatives represent an important class of oxygen-containing heterocyclic com­pounds that continue to attract significant attention in medicinal and synthetic organic chemistry because of their wide range of biological and pharmacological activities, which include anti­microbial, anti­oxidant, anti-inflammatory, anti­viral, anti­diabetic and anti­cancer properties (Chaudary *et al.*, 2022 ▸; Kativar *et al.*, 2022 ▸; Patel *et al.*, 2025 ▸). Moreover, the chromene scaffold is considered a privileged structural motif that is frequently encountered in natural products and bioactive synthetic mol­ecules, because of its importance in drug discovery and medicinal chemistry research (Raj *et al.*, 2013 ▸; Welsch *et al.*, 2010 ▸).
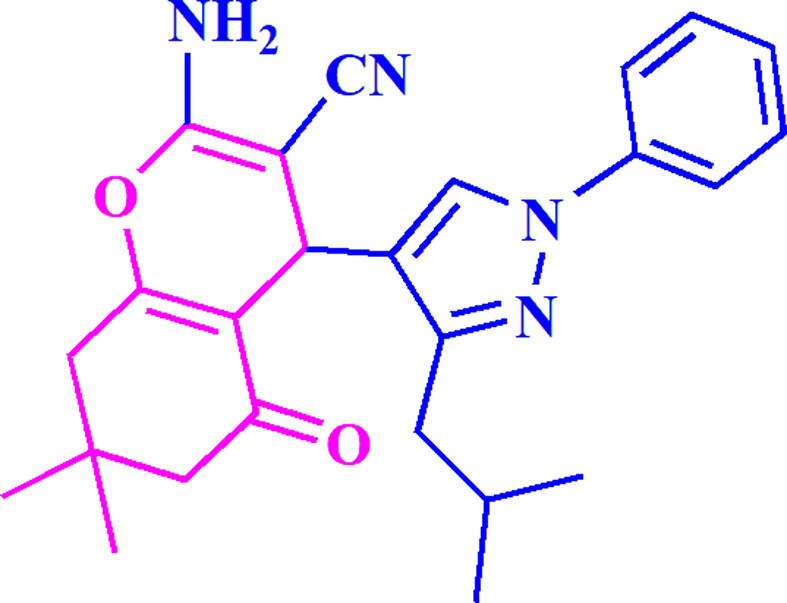


Among these systems, 2-amino-4*H*-chromene derivatives have received particular inter­est because of their facile syn­thetic accessibility and biological potential. They are widely used as key building blocks for the synthesis of more com­plex heterocyclic systems. Thus, combining the chromene framework with other biologically active heterocycles, such as pyrazole rings, has been shown to generate hybrid structures with enhanced pharmacological properties related to synergistic electronic and structural effects (Santos *et al.*, 2017 ▸; Hebishy *et al.*, 2022 ▸).

From a synthetic point of view, multicom­ponent reactions are among the most efficient and widely used strategies for constructing chromene derivatives. These methods are attractive because they are operationally simple, environmentally friendly and provide high atom economy with good yields, making them suitable for the rapid generation of diverse chromene libraries (Kidwai *et al.*, 2005 ▸; Abdallah *et al.*, 2022 ▸; Abdallah *et al.*, 2023*a* ▸; Abdallah *et al.*, 2023*b* ▸).

In this context, our laboratory (the Elgemeie group) has recently contributed to the field of heterocyclic chemistry, particularly in the development of new synthetic methodologies and biologically active nitro­gen- and oxygen-containing heterocyclic systems, including pyrazole-based frameworks (Elboshi *et al.*, 2024 ▸).

**Figure 1:**
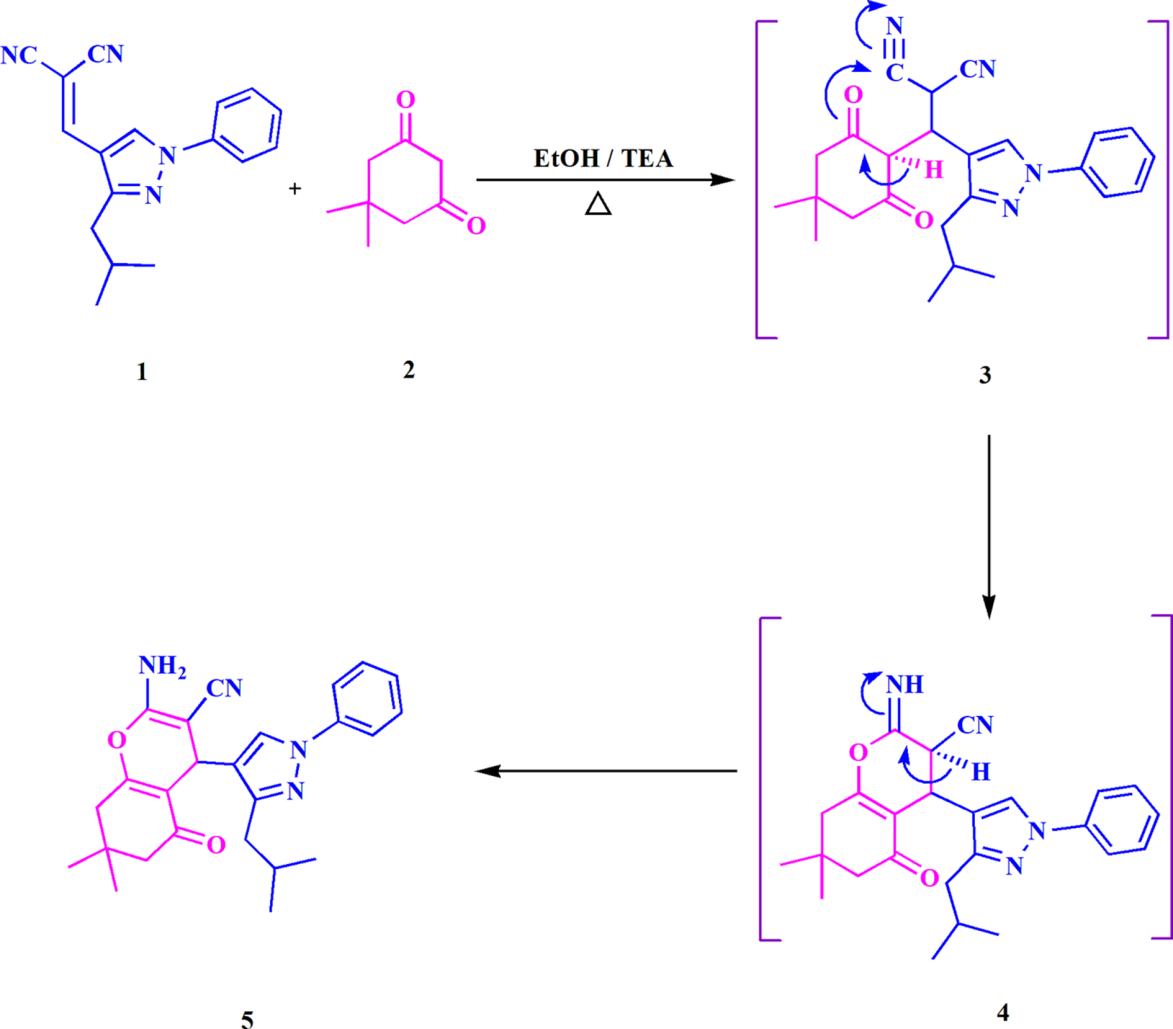
The synthesis of com­pound **5** (TEA = tri­ethyl­amine).

The reaction (Fig. 1 ▸) between the pyrazole methyl­enemalono­nitrile derivative **1** and dimedone **2** was carried out in refluxing ethanol in the presence of a few drops of tri­ethyl­amine with continuous stirring. After 2 h, the desired chromene derivative **5** was isolated. The synthesis probably proceeds through initial formation of the inter­mediate Michael addition product **3**, followed by intra­molecular cyclization to **4**, finally leading to the product **5**. The structure of **5** was deduced on the basis of IR and ^1^H NMR spectroscopic analyses. The IR spectrum displayed char­acteristic absorption bands at 3360 cm^−1^ corresponding to the amino group, 2215 cm^−1^ attributed to the nitrile functionality and 1741 cm^−1^ assigned to the carbonyl group. Additional confirmation was obtained from the ^1^H NMR spectrum. A characteristic singlet appeared at δ 4.27 ppm and was assigned to the methine proton of the chromene ring system. Moreover, the disappearance of the vinylic proton signal, present in the starting material **1**, indicated successful cyclization and formation of the chromene framework. The spectrum also showed the appearance of a new signal at δ 6.94 ppm corresponding to the amino group (NH_2_), providing further evidence for the formation of the target com­pound. Unambiguous confirmation of the nature of **5** was provided by the X-ray structure determination, pre­sent­ed here.

## 2. Structural commentary

**Figure 2:**
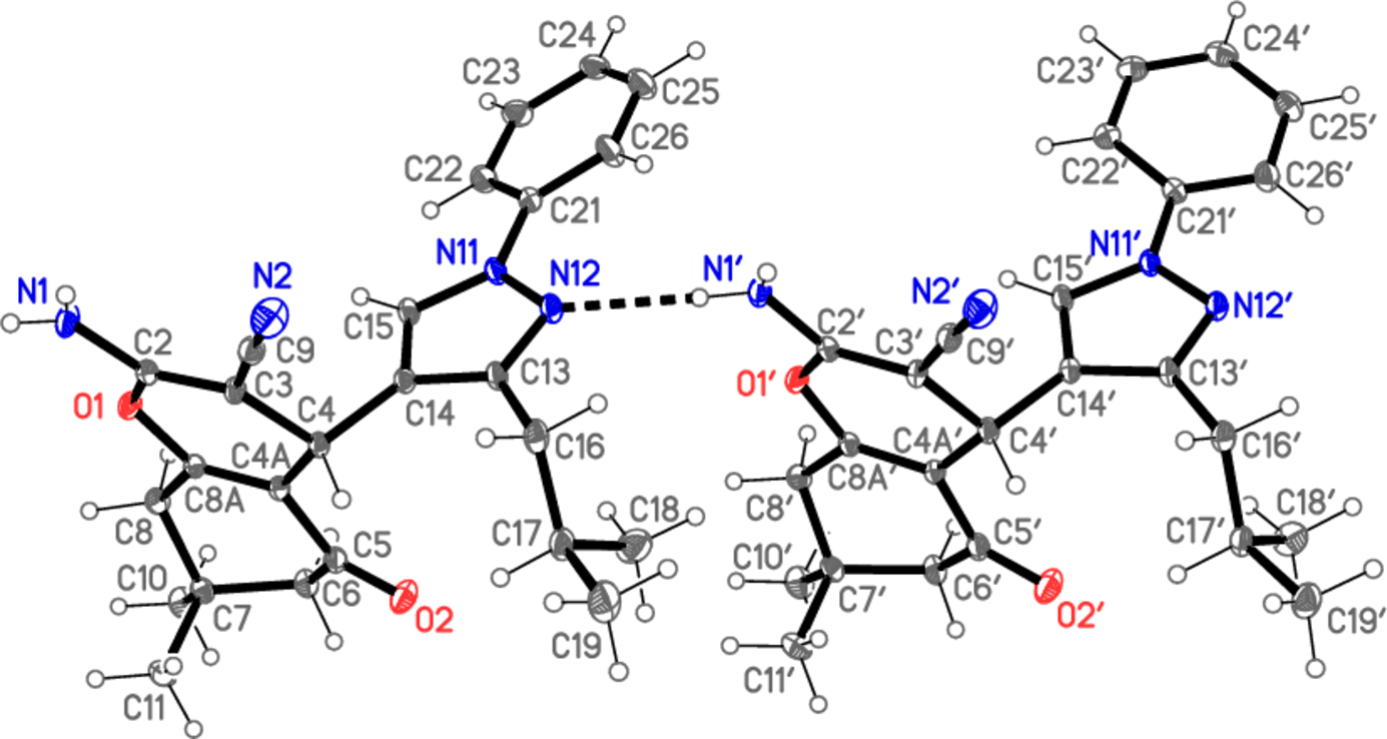
The asymmetric unit of com­pound **5** in the crystal. Displacement ellipsoids correspond to 50% probability levels. The dashed line indicates a classical hy­dro­gen bond.

**Figure 3:**
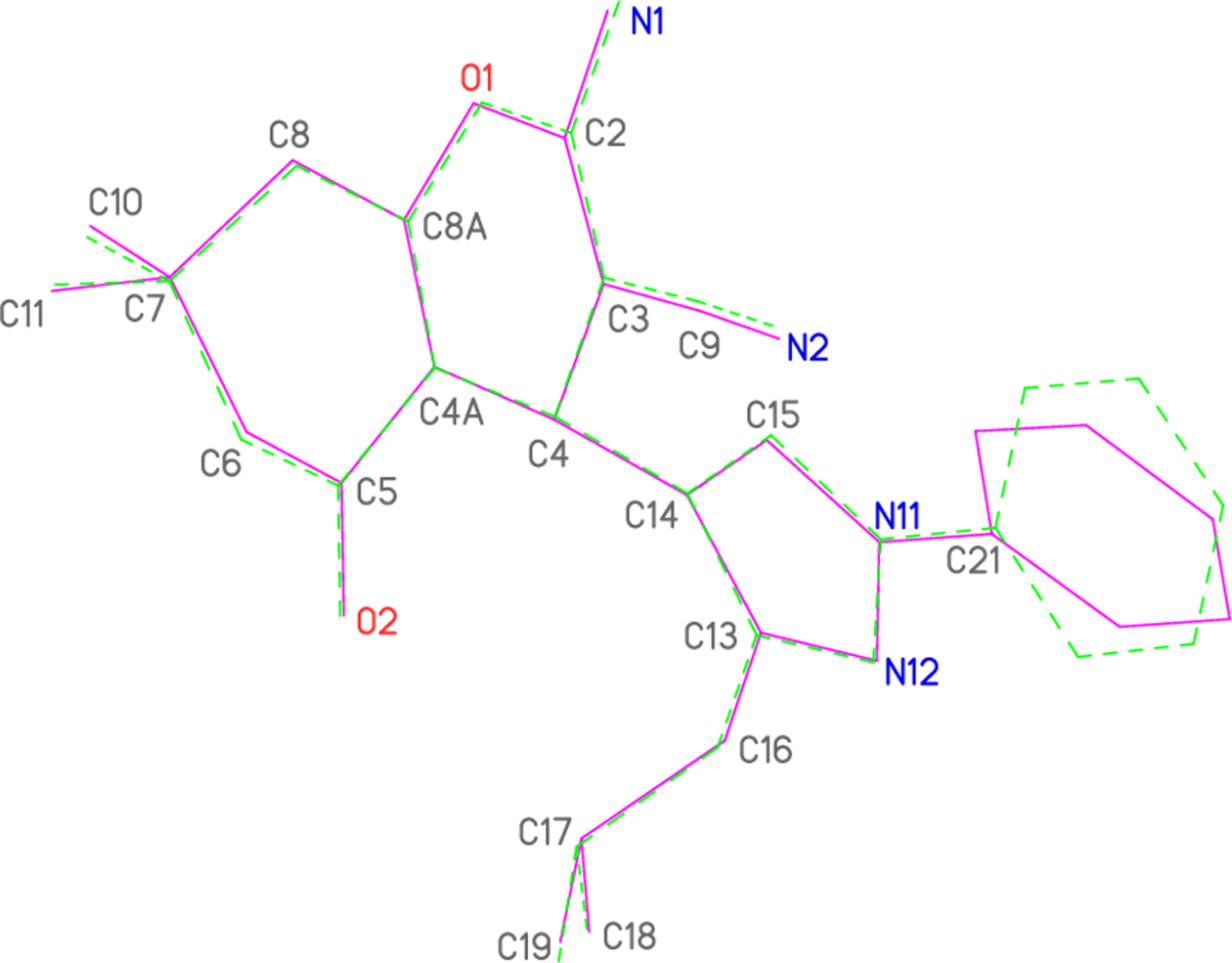
A least-squares fit of the two independent mol­ecules of **5**, showing the different orientations of the ring at C21. The second mol­ecule is shown with green dashed bonds. Fitted atoms of the first mol­ecule (magenta bonds) are labelled.

The structure of com­pound **5** in the crystal is shown in Fig. 2 ▸; there are two mol­ecules in the asymmetric unit, related approximately by the translation (*x* + 1, *y* + 1, *z*), and differing mainly in the orientation of phenyl ring C21–26. Atom names of the second mol­ecule are distinguished by primes (′). Both independent mol­ecules have the configuration *S* at C4, the only stereogenic C atom (although the overall constitution is of course a racemate). Selected mol­ecular dimensions are given in Table 1 ▸. A least-squares fit of both mol­ecules, considering all non-H atoms except C22–26 (Fig. 3 ▸; *cf*. torsion angles in Table 1 ▸) gives an r.m.s. deviation (rmsd) of 0.11 Å.

In the (substanti­ally modified) chromene ring systems, the formal double bonds C2=C3 and C4*A*=C8*A* are by far the shortest C—C bonds. The *sp*^3^ hybridization of C4 means that the ring angle at this atom is substanti­ally more acute than other angles of the oxo ring. This ring displays a flattened boat conformation, with O1 lying 0.082 (1) and C4 0.156 (1) Å to the same side of the plane of the other four atoms (rmsd 0.004 Å); in the second mol­ecule, the corresponding values are 0.090 (1) and 0.193 (1) Å, rmsd 0.003 Å. The second ring is approximately a flattened envelope, with C7 lying 0.588 (1) Å out of the plane of the other five atoms (rmsd 0.061 Å); in the second mol­ecule, C7′ lies 0.596 (1) Å out of plane, rmsd 0.057 Å. The bond angles of this ring, with formal *sp*^3^ hybridization at C6, C7 and C8, but *sp*^2^ at C4*A*, C5 and C8*A*, vary considerably, with the widest at C8*A* and the narrowest at C7 [126.01 (8) and 108.76 (7)°, respectively]. Other mol­ecular dimensions (*e.g.* of the pyrazole rings) may be regarded as unexceptional.

The inter­planar angles between the pyrazole and phenyl rings are 14.66 (6)° for mol­ecule 1 (rmsd of individual rings: 0.004 and 0.005 Å) and 14.67 (7)° (0.003 and 0.007 Å) for mol­ecule 2. However, these figures mask the fact that the mutual rotation is in opposite senses for the two mol­ecules (see above and Fig. 3 ▸), so that the relevant torsion angles have opposite signs. The torsion angles about the bond C4—C14 (and C4′—C14′), defining the relative orientation of the oxo and pyrazole rings, differ by *ca* 4–6°.

## 3. Supra­molecular features

**Figure 4:**
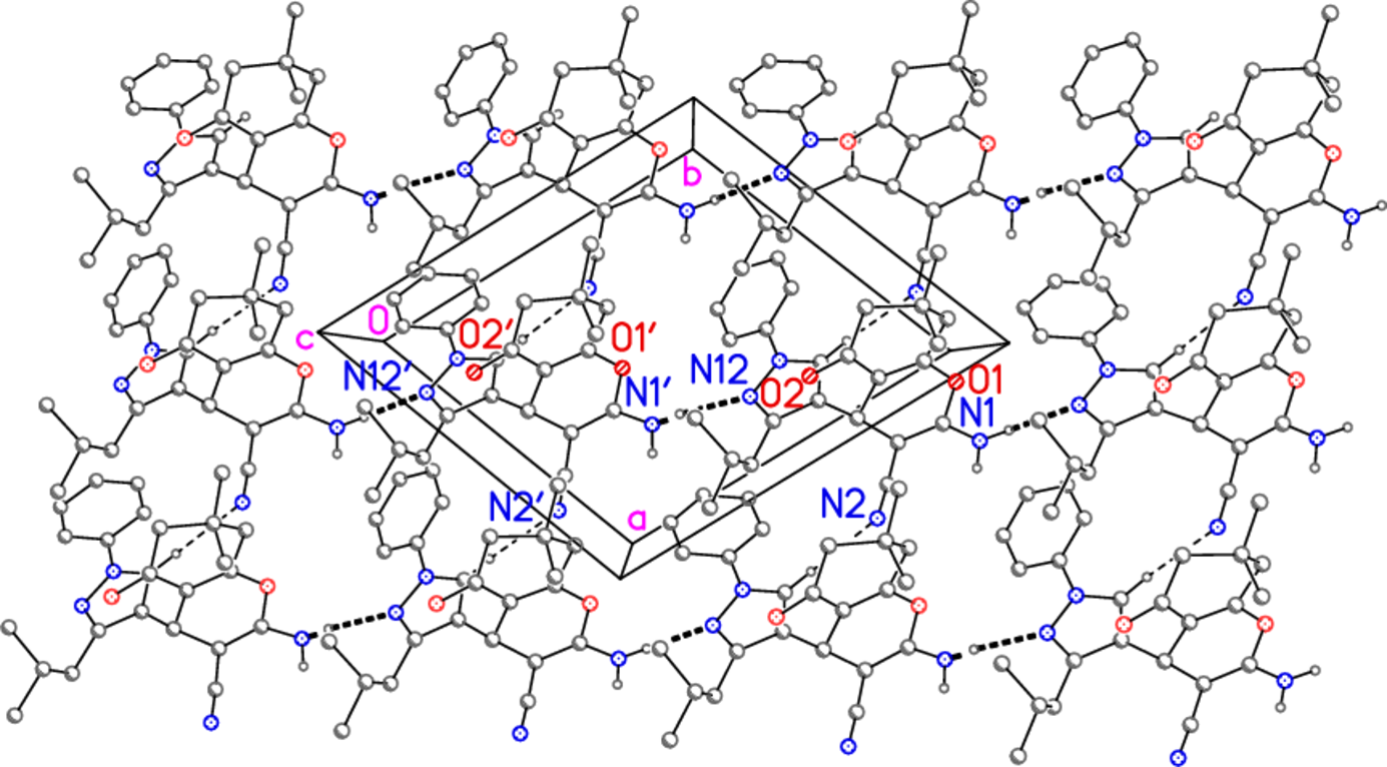
Packing diagram of com­pound **5**, viewed parallel to the *c* axis in the region *z* ≃ 3/4. Mol­ecules are connected by classical hy­dro­gen bonds (thick dashed lines) to form ribbons parallel to [110] (horizontal; three such ribbons are shown). Ribbons are connected by weak hy­dro­gen bonds C—H⋯N≡C (thin dashed lines) and perhaps also by C—H⋯π contacts (not drawn explicitly; see text). The alternating orientations of the rings C21–26 and C21′–26′ are clearly recognizable (*e.g.* at the top of the diagram)..

Hydrogen bonds are listed in Table 2 ▸. The mol­ecules of **5** are connected by classical hy­dro­gen bonds N1′—H01′⋯N12 and N1—H01⋯N12′ (*x* + 1, *y* + 1, *z*) to form a ribbon of alternating independent mol­ecules parallel to [110]. Three such ribbons are shown, running horizontally, in Fig. 4 ▸. Adjacent ribbons are joined by weak hy­dro­gen bonds from the pyrazole rings to the nitrile N atoms, namely C15—H15⋯N2′ (*x*, *y* + 1, *z*) and C15′—H15′⋯N2 (*x* − 1, *y*, *z*) to form a layer structure parallel to the *ab* plane in the region *z* ≃ 3/4 (a further such layer, but inverted, occupies the region *z* ≃ 1/4). Layers are connected in the third dimension by C_meth­yl_—H⋯O=C contacts (not shown in Fig. 4 ▸). It is noteworthy that the potential hy­dro­gen-bond donors H02 and H02′ form neither classical nor weak hy­dro­gen bonds, but instead are directed towards phenyl rings, with N1—H02⋯*Cg*(C21–26) = 2.83 and N1′—H02′⋯*Cg*(C21′–26′) = 2.98 Å (operator: *x* + 1, *y*, *z* in both cases). The H⋯C distances however vary considerably, the shortest for each H atom being H02⋯C24 = 2.74 (2) and H02′⋯C25′ = 2.53 (2) Å. These contacts can be recognized between adjacent ribbons in Fig. 4 ▸, but are not drawn explicitly.

## 4. Database survey

Searches were conducted using the Cambridge Structural Database (CSD, Version 6.01, update November 2025; Groom *et al.*, 2016 ▸) and the *ConQuest* routine (Version 2025.3.1; Bruno *et al.*, 2002 ▸).

A search for the 5,6,7,8-tetra­hydro-4*H*-chromene skeleton, allowing any substitution but omitting further annelated rings, gave 387 hits. Restricting the search to 2-amino-3-carbo­nitrile derivatives with a C and an H substituent at C4 still gave 148 hits, showing that this is a much-studied group of com­pounds. Finally, restricting the non-H substituent at C4 to a non-annelated five-membered ring gave only 6 hits; these involved 1,2-thia­zole or 1,2-oxazole (refcodes PUFPUF and PUFQAM; Potkin *et al.*, 2024 ▸), thio­phene (QEFVUW and UGUPUL; Zamisa *et al.*, 2022 ▸; Nyapola *et al.*, 2024 ▸) or furan (VAMRIO and XUYCUQ; Kalantari *et al.*, 2021 ▸; Song *et al.*, 2010 ▸) derivatives.

Alternatively, restricting the substitution at C7 to dimethyl, while still ruling out further annelation, gave just one hit, also a 2-amino-3-carbo­nitrile derivative, namely, 2-amino-4-(4-meth­oxy­phen­yl)-6,6-dimethyl-5-oxo-5,6,7,8-tetra­hydro-4*H*-1-benzo­pyran-3-carbo­nitrile (OCEWUS; Djedouani & Bourzami, 2021 ▸).

## 5. Synthesis and crystallization

The synthesis, including a proposed mechanism, is shown in Fig. 1 ▸. To a solution of 2-[(3-isobutyl-1-phenyl-1*H*-pyrazol-4-yl)methyl­idene]malono­nitrile, **1** (1 mmol), in ethanol (20 ml) was added 5,5-di­methyl­cyclo­hexane-1,3-dione, **2** (1 mmol). Subsequently, a few drops of tri­ethyl­amine were added and the reaction mixture was heated under reflux for 2 h. The progress of the reaction was monitored by thin-layer chromatography (TLC). After com­pletion of the reaction, the precipitate was collected by hot filtration, washed with ethanol, dried and recrystallized from ethanol to afford colourless crystals of the desired product **5** (yield: 0.395 g, 95%; m.p. 482–484 K). IR (cm^−1^) : ν 3360 (NH_2_), 2949 (CH_3_), 2215 (C≡N), 1741 (C=O); ^1^H NMR (400 MHz, DMSO-*d*_6_): δ ppm 0.99–1.08 (*m*, 12H, 4 × CH_3_), 2.07–2.14 (*m*, 1H, CH), 2.18 (*d*, *J* = 14 Hz, 2H, chromene-CH_2_), 2.54–2.59 (*m*, 2H, chromene-CH_2_), 4.27 (*s*, 1H, chromene-CH), 6.94 (*s*, D_2_O-exchangeable, 2H, NH_2_), 7.22 (*t*, *J* = 7.6 Hz, 1H, Ar-H), 7.43 (*t*, *J* = 8.4 Hz, 2H, Ar-H), 7.76 (*d*, *J* = 8.0 Hz, 2H, Ar-H), 8.15 (*s*, 1H, pyrazole-H). Analysis calculated (%) for C_25_H_28_N_4_O_2_: C 72.09, H 6.78, N 13.45; found: C 72.10, H 6.80; N 13.47.

## 6. Refinement

Details of data collection and structure refinement are summarized in Table 3 ▸.

The structure was refined as a two-com­ponent non-merohedral twin (involving 180° rotation about c*) using the ‘HKLF 5’ method. The relative contribution of the smaller twin com­ponent refined to 0.4461 (6). The dataset com­prises all overlapped and non-overlapped reflections from both com­ponents, so that the number of reflections given in Table 3 ▸ should be inter­preted with caution. *R*_int_ is meaningless because equivalents are merged during the data reduction of twins with *CrysAlis PRO* (Rigaku OD, 2026 ▸).

Atom names of the second independent mol­ecule are distinguished by primes (′). H atoms of the NH_2_ groups were refined freely. The methyl groups were refined as idealized rigid groups, with C—H 0.98 Å and H—C—H 109.5°, allowed to rotate but not tip (AFIX 137). Other H atoms were included using a riding model starting from calculated positions, with C—H = 1.00, 0.99 and 0.95 Å for methine, methyl­ene and aromatic H atoms, respectively. The *U*_eq_(H) values were fixed at 1.5*U*_eq_ of the parent C atoms for the methyl groups and at 1.2*U*_eq_ for the other H atoms. Three badly-fitting reflections with Δ/σ > 10 were omitted from the refinement.

*CheckCIF* suggests missing translational symmetry between the two independent mol­ecules (translation *x* + 1, *y* + 1, *z*), but this is only approximate (see, for example, the differing orientations of the phenyl groups for the two mol­ecules, as discussed above).

## Supplementary Material

Crystal structure: contains datablock(s) I, global. DOI: 10.1107/S2056989026006973/yz2082sup1.cif

Structure factors: contains datablock(s) I. DOI: 10.1107/S2056989026006973/yz2082Isup2.hkl

Supporting information file. DOI: 10.1107/S2056989026006973/yz2082Isup3.cml

CCDC reference: 2570429

Additional supporting information:  crystallographic information; 3D view; checkCIF report

## Figures and Tables

**Table 1:** Selected geometric parameters (Å, °)

C2—C3	1.3623 (12)	C2′—C3′	1.3629 (12)
C4*A*—C8*A*	1.3404 (12)	C4*A*′—C8*A*′	1.3446 (12)
N11—C15	1.3644 (12)	N11′—C15′	1.3627 (12)
N11—N12	1.3644 (11)	N11′—N12′	1.3631 (11)
N12—C13	1.3368 (11)	N12′—C13′	1.3371 (11)
C13—C14	1.4176 (12)	C13′—C14′	1.4183 (12)
C14—C15	1.3800 (12)	C14′—C15′	1.3809 (12)
			
C4*A*—C4—C3	108.28 (7)	C4*A*′—C4′—C3′	108.14 (7)
C6—C7—C8	108.76 (7)	C6′—C7′—C8′	108.67 (7)
C4*A*—C8*A*—C8	126.01 (8)	C4*A*′—C8*A*′—C8′	125.76 (8)
			
C3—C4—C14—C15	−72.98 (11)	C3′—C4′—C14′—C15′	−68.38 (11)
C3—C4—C14—C13	103.58 (10)	C3′—C4′—C14′—C13′	109.27 (10)
N12—N11—C21—C22	166.67 (8)	N12′—N11′—C21′—C22′	−164.36 (9)
N12—N11—C21—C26	−14.27 (13)	N12′—N11′—C21′—C26′	15.71 (13)

**Table 2:** Hydrogen-bond geometry (Å, °)

*D*—H⋯*A*	*D*—H	H⋯*A*	*D*⋯*A*	*D*—H⋯*A*
N1′—H01′⋯N12	0.878 (17)	2.115 (17)	2.9895 (11)	173.5 (14)
N1—H01⋯N12′^i^	0.868 (16)	2.145 (16)	3.0071 (11)	172.0 (15)
C15—H15⋯N2′^ii^	0.95	2.41	3.3622 (13)	176
C15′—H15′⋯N2^iii^	0.95	2.43	3.3816 (13)	180
C11—H11*A*⋯O2′^iv^	0.98	2.47	3.3965 (12)	157
C11′—H11*D*⋯O2^iv^	0.98	2.59	3.5046 (13)	155

Symmetry codes: (i) 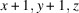; (ii) 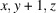; (iii) 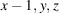; (iv) 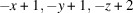.

**Table 3:** Experimental details

Crystal data	
Chemical formula	C_25_H_28_N_4_O_2_
*M*_r_	416.51
Crystal system, space group	Triclinic, *P* 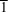
Temperature (K)	100
*a*, *b*, *c* (Å)	10.6988 (4), 12.0191 (4), 18.3578 (6)
α, β, γ (°)	83.864 (3), 80.248 (3), 69.426 (3)
*V* (Å^3^)	2175.26 (14)
*Z*	4
Radiation type	Mo *K*α
μ (mm^−1^)	0.08
Crystal size (mm)	0.18 × 0.12 × 0.05

Data collection	
Diffractometer	Bruker XtaLAB Synergy
Absorption correction	Multi-scan (*CrysAlis PRO*; Rigaku OD, 2026 ▸)
*T*_min_, *T*_max_	0.860, 1.000
No. of measured, independent and observed [*I* > 2σ(*I*)] reflections	24964, 24964, 20260
*R*_int_	See *Refinement* section
θ values (°)	θ_max_ = 38.6, θ_min_ = 2.1
(sin θ/λ)_max_ (Å^−1^)	0.877

Refinement	
*R*[*F*^2^ > 2σ(*F*^2^)], *wR*(*F*^2^), *S*	0.049, 0.133, 1.04
No. of reflections	24964
No. of parameters	584
H-atom treatment	H atoms treated by a mixture of independent and constrained refinement
Δρ_max_, Δρ_min_ (e Å^−3^)	0.50, −0.29

Computer programs: *CrysAlis PRO* (Rigaku OD, 2026 ▸), *SHELXT* (Sheldrick, 2015*a* ▸), *SHELXL2019* (Sheldrick, 2015*b* ▸), *XP* (Bruker, 1998 ▸) and *publCIF* (Westrip, 2010 ▸).

## supplementary crystallographic information

### 2-Amino-4-(3-isobutyl-1-phenyl-1H-pyrazol-4-yl)-7,7-dimethyl-5-oxo-5,6,7,8-tetrahydro-4H-chromene-3-carbonitrile Crystal data

**Table:**

C_25_H_28_N_4_O_2_	*Z* = 4
*M**_r_* = 416.51	*F*(000) = 888
Triclinic, *P*1	*D*_x_ = 1.272 Mg m^−^^3^
*a* = 10.6988 (4) Å	Mo *K*α radiation, λ = 0.71073 Å
*b* = 12.0191 (4) Å	Cell parameters from 47190 reflections
*c* = 18.3578 (6) Å	θ = 2.2–39.6°
α = 83.864 (3)°	µ = 0.08 mm^−^^1^
β = 80.248 (3)°	*T* = 100 K
γ = 69.426 (3)°	Block, colourless
*V* = 2175.26 (14) Å^3^	0.18 × 0.12 × 0.05 mm

### 2-Amino-4-(3-isobutyl-1-phenyl-1H-pyrazol-4-yl)-7,7-dimethyl-5-oxo-5,6,7,8-tetrahydro-4H-chromene-3-carbonitrile Data collection

**Table:**

Bruker XtaLAB Synergy diffractometer	24964 measured reflections
Radiation source: micro-focus sealed X-ray tube, PhotonJet (Mo) X-ray Source	24964 independent reflections
Mirror monochromator	20260 reflections with *I* > 2σ(*I*)
Detector resolution: 10.0000 pixels mm^-1^	θ_max_ = 38.6°, θ_min_ = 2.1°
ω scans	*h* = −18→18
Absorption correction: multi-scan (CrysAlis PRO; Rigaku OD, 2026)	*k* = −21→21
*T*_min_ = 0.860, *T*_max_ = 1.000	*l* = −32→32

### 2-Amino-4-(3-isobutyl-1-phenyl-1H-pyrazol-4-yl)-7,7-dimethyl-5-oxo-5,6,7,8-tetrahydro-4H-chromene-3-carbonitrile Refinement

**Table:**

Refinement on *F*^2^	Primary atom site location: dual
Least-squares matrix: full	Hydrogen site location: mixed
*R*[*F*^2^ > 2σ(*F*^2^)] = 0.049	H atoms treated by a mixture of independent and constrained refinement
*wR*(*F*^2^) = 0.133	*w* = 1/[σ^2^(*F*_o_^2^) + (0.0823*P*)^2^] where *P* = (*F*_o_^2^ + 2*F*_c_^2^)/3
*S* = 1.04	(Δ/σ)_max_ = 0.001
24964 reflections	Δρ_max_ = 0.50 e Å^−^^3^
584 parameters	Δρ_min_ = −0.29 e Å^−^^3^
0 restraints	

### 2-Amino-4-(3-isobutyl-1-phenyl-1H-pyrazol-4-yl)-7,7-dimethyl-5-oxo-5,6,7,8-tetrahydro-4H-chromene-3-carbonitrile Fractional atomic coordinates and isotropic or equivalent isotropic displacement parameters (Å^2^)

**Table:**

	*x*	*y*	*z*	*U*_iso_*/*U*_eq_	
O1	1.03615 (7)	0.87672 (6)	0.73010 (4)	0.01311 (11)	
C2	1.12004 (9)	0.77845 (7)	0.69375 (5)	0.01168 (13)	
C3	1.09881 (8)	0.67220 (7)	0.70413 (5)	0.01130 (13)	
C4	0.97828 (8)	0.65456 (7)	0.75463 (5)	0.01088 (13)	
H4	1.012593	0.584062	0.789162	0.013*	
C4A	0.91003 (9)	0.76258 (7)	0.80014 (5)	0.01118 (13)	
C5	0.81237 (9)	0.75435 (8)	0.86584 (5)	0.01327 (14)	
C6	0.72387 (9)	0.86867 (8)	0.90112 (5)	0.01458 (15)	
H6A	0.685693	0.849908	0.951979	0.018*	
H6B	0.647854	0.908341	0.872649	0.018*	
C7	0.80005 (9)	0.95482 (8)	0.90446 (5)	0.01311 (14)	
C8	0.86883 (9)	0.97571 (8)	0.82595 (5)	0.01387 (14)	
H8A	0.800163	1.031625	0.797127	0.017*	
H8B	0.936126	1.013455	0.829478	0.017*	
C8A	0.93736 (9)	0.86397 (8)	0.78557 (5)	0.01147 (13)	
C9	1.18878 (9)	0.57506 (8)	0.66364 (5)	0.01410 (14)	
C10	0.70049 (10)	1.07310 (9)	0.93309 (6)	0.01883 (17)	
H10A	0.656800	1.059625	0.982990	0.028*	
H10B	0.631947	1.107160	0.900028	0.028*	
H10C	0.748914	1.128338	0.934599	0.028*	
C11	0.90628 (10)	0.90137 (9)	0.95686 (5)	0.01641 (16)	
H11A	0.862044	0.885605	1.006165	0.025*	
H11B	0.952675	0.957596	0.960027	0.025*	
H11C	0.971956	0.826772	0.937852	0.025*	
N11	0.72968 (8)	0.65100 (7)	0.63907 (4)	0.01300 (13)	
N12	0.77313 (8)	0.53324 (7)	0.66106 (4)	0.01358 (13)	
C13	0.86526 (9)	0.52040 (8)	0.70515 (5)	0.01247 (14)	
C14	0.88152 (9)	0.63071 (7)	0.71208 (5)	0.01147 (13)	
C15	0.79426 (9)	0.71150 (8)	0.66850 (5)	0.01307 (14)	
H15	0.781511	0.793932	0.660488	0.016*	
C16	0.93578 (9)	0.40083 (8)	0.73944 (5)	0.01562 (15)	
H16A	1.033842	0.386946	0.731277	0.019*	
H16B	0.921007	0.339576	0.713312	0.019*	
C17	0.89145 (10)	0.38313 (9)	0.82300 (6)	0.01776 (17)	
H17	0.912874	0.441064	0.849656	0.021*	
C18	0.74040 (11)	0.40549 (11)	0.84093 (7)	0.0260 (2)	
H18A	0.690560	0.486101	0.822925	0.039*	
H18B	0.715587	0.397308	0.894617	0.039*	
H18C	0.717992	0.347451	0.816784	0.039*	
C19	0.97173 (13)	0.25766 (10)	0.84956 (8)	0.0284 (2)	
H19A	0.952334	0.199518	0.823822	0.043*	
H19B	0.946136	0.247012	0.902999	0.043*	
H19C	1.068321	0.245788	0.838950	0.043*	
C21	0.63208 (9)	0.69587 (8)	0.58987 (5)	0.01323 (14)	
C22	0.56792 (10)	0.81835 (8)	0.57979 (5)	0.01687 (16)	
H22	0.589748	0.872145	0.605423	0.020*	
C23	0.47161 (10)	0.86106 (9)	0.53182 (5)	0.01902 (17)	
H23	0.427163	0.944361	0.525138	0.023*	
C24	0.43990 (10)	0.78300 (9)	0.49364 (5)	0.01865 (17)	
H24	0.373450	0.812711	0.461390	0.022*	
C25	0.50581 (11)	0.66156 (10)	0.50290 (6)	0.02197 (19)	
H25	0.485252	0.608047	0.476339	0.026*	
C26	0.60208 (11)	0.61742 (9)	0.55093 (6)	0.02040 (18)	
H26	0.647045	0.534104	0.557058	0.024*	
O2	0.80446 (8)	0.65736 (7)	0.88973 (4)	0.01929 (14)	
N1	1.22003 (9)	0.80417 (8)	0.64810 (5)	0.01663 (14)	
H01	1.2362 (15)	0.8692 (14)	0.6514 (9)	0.021 (4)*	
H02	1.2847 (17)	0.7476 (16)	0.6228 (10)	0.035 (5)*	
N2	1.25994 (10)	0.49270 (8)	0.63172 (5)	0.02255 (18)	
O1'	0.51350 (7)	0.38781 (6)	0.73024 (4)	0.01354 (11)	
C2'	0.60315 (9)	0.29023 (8)	0.69527 (5)	0.01174 (13)	
C3'	0.59363 (9)	0.17973 (7)	0.71029 (5)	0.01188 (13)	
C4'	0.47740 (9)	0.15715 (7)	0.76234 (5)	0.01175 (13)	
H4'	0.515849	0.088341	0.797219	0.014*	
C4A'	0.40446 (9)	0.26552 (8)	0.80681 (5)	0.01190 (13)	
C5'	0.31341 (9)	0.25433 (8)	0.87492 (5)	0.01413 (14)	
C6'	0.22061 (9)	0.36745 (8)	0.91023 (5)	0.01551 (15)	
H6'A	0.188777	0.348302	0.962284	0.019*	
H6'B	0.140759	0.401462	0.884021	0.019*	
C7'	0.28899 (9)	0.46115 (8)	0.90849 (5)	0.01372 (14)	
C8'	0.34790 (10)	0.48320 (8)	0.82794 (5)	0.01479 (15)	
H8'A	0.273601	0.533318	0.800627	0.018*	
H8'B	0.410198	0.527719	0.827748	0.018*	
C8A'	0.42197 (9)	0.37097 (8)	0.78865 (5)	0.01198 (13)	
C9'	0.68982 (9)	0.08237 (8)	0.67210 (5)	0.01403 (14)	
C10'	0.18431 (11)	0.57673 (9)	0.93786 (6)	0.02038 (18)	
H10D	0.147481	0.562286	0.989187	0.031*	
H10E	0.111258	0.604822	0.907374	0.031*	
H10F	0.227065	0.637160	0.935860	0.031*	
C11'	0.40197 (10)	0.41579 (9)	0.95718 (5)	0.01834 (17)	
H11D	0.363728	0.401533	1.008245	0.028*	
H11E	0.445565	0.475482	0.955834	0.028*	
H11F	0.468741	0.341393	0.938564	0.028*	
N11'	0.23381 (8)	0.13942 (7)	0.64850 (4)	0.01237 (12)	
N12'	0.27363 (8)	0.02430 (7)	0.67587 (4)	0.01332 (13)	
C13'	0.36444 (9)	0.01589 (8)	0.71992 (5)	0.01276 (14)	
C14'	0.38324 (9)	0.12666 (8)	0.72139 (5)	0.01173 (13)	
C15'	0.29889 (9)	0.20282 (8)	0.67452 (5)	0.01320 (14)	
H15'	0.288210	0.284238	0.662630	0.016*	
C16'	0.43264 (9)	−0.10117 (8)	0.75833 (5)	0.01475 (15)	
H16C	0.530516	−0.114623	0.752041	0.018*	
H16D	0.420694	−0.164959	0.733255	0.018*	
C17'	0.38266 (9)	−0.11354 (8)	0.84158 (5)	0.01480 (15)	
H17'	0.406063	−0.056508	0.867963	0.018*	
C18'	0.23033 (10)	−0.08467 (10)	0.85660 (6)	0.02166 (19)	
H18D	0.185330	−0.002565	0.839034	0.032*	
H18E	0.202154	−0.093708	0.909902	0.032*	
H18F	0.205578	−0.139190	0.830536	0.032*	
C19'	0.45446 (11)	−0.23934 (9)	0.87109 (6)	0.02051 (18)	
H19D	0.431693	−0.296340	0.846160	0.031*	
H19E	0.425742	−0.246242	0.924482	0.031*	
H19F	0.552168	−0.256378	0.861634	0.031*	
C21'	0.13891 (9)	0.17986 (8)	0.59760 (5)	0.01348 (14)	
C22'	0.12388 (11)	0.28727 (9)	0.55669 (5)	0.01822 (17)	
H22'	0.176213	0.334178	0.563006	0.022*	
C23'	0.03152 (11)	0.32499 (10)	0.50659 (6)	0.02094 (18)	
H23'	0.020888	0.398243	0.478748	0.025*	
C24'	−0.04542 (11)	0.25734 (10)	0.49657 (5)	0.01992 (18)	
H24'	−0.108510	0.284108	0.462316	0.024*	
C25'	−0.02916 (10)	0.15003 (9)	0.53721 (6)	0.01906 (17)	
H25'	−0.079922	0.102393	0.529701	0.023*	
C26'	0.06093 (10)	0.11146 (9)	0.58894 (6)	0.01719 (16)	
H26'	0.069140	0.039534	0.617928	0.021*	
O2'	0.31355 (8)	0.15590 (7)	0.90040 (4)	0.02190 (15)	
N1'	0.69523 (9)	0.32195 (8)	0.64594 (5)	0.01595 (14)	
H01'	0.7113 (15)	0.3871 (15)	0.6517 (9)	0.027 (4)*	
H02'	0.7673 (18)	0.2640 (16)	0.6248 (10)	0.037 (5)*	
N2'	0.76661 (10)	−0.00011 (8)	0.64231 (5)	0.02121 (17)	

### 2-Amino-4-(3-isobutyl-1-phenyl-1H-pyrazol-4-yl)-7,7-dimethyl-5-oxo-5,6,7,8-tetrahydro-4H-chromene-3-carbonitrile Atomic displacement parameters (Å^2^)

**Table:**

	*U*^11^	*U*^22^	*U*^33^	*U*^12^	*U*^13^	*U*^23^
O1	0.0164 (3)	0.0098 (2)	0.0141 (3)	−0.0066 (2)	0.0010 (2)	−0.0021 (2)
C2	0.0137 (3)	0.0110 (3)	0.0121 (3)	−0.0063 (3)	−0.0020 (3)	−0.0009 (2)
C3	0.0119 (3)	0.0096 (3)	0.0133 (3)	−0.0047 (3)	−0.0010 (3)	−0.0015 (2)
C4	0.0117 (3)	0.0096 (3)	0.0128 (3)	−0.0052 (3)	−0.0021 (2)	−0.0007 (2)
C4A	0.0132 (3)	0.0100 (3)	0.0118 (3)	−0.0054 (3)	−0.0019 (2)	−0.0011 (2)
C5	0.0136 (3)	0.0142 (3)	0.0136 (3)	−0.0067 (3)	−0.0012 (3)	−0.0015 (3)
C6	0.0128 (3)	0.0151 (4)	0.0167 (3)	−0.0056 (3)	−0.0009 (3)	−0.0035 (3)
C7	0.0136 (3)	0.0125 (3)	0.0136 (3)	−0.0042 (3)	−0.0017 (3)	−0.0034 (3)
C8	0.0173 (4)	0.0108 (3)	0.0142 (3)	−0.0053 (3)	−0.0015 (3)	−0.0029 (3)
C8A	0.0142 (3)	0.0104 (3)	0.0113 (3)	−0.0058 (3)	−0.0021 (2)	−0.0006 (2)
C9	0.0154 (4)	0.0128 (3)	0.0150 (3)	−0.0065 (3)	−0.0013 (3)	−0.0002 (3)
C10	0.0189 (4)	0.0149 (4)	0.0216 (4)	−0.0038 (3)	−0.0006 (3)	−0.0071 (3)
C11	0.0163 (4)	0.0199 (4)	0.0142 (3)	−0.0065 (3)	−0.0038 (3)	−0.0024 (3)
N11	0.0155 (3)	0.0107 (3)	0.0157 (3)	−0.0065 (2)	−0.0059 (2)	−0.0003 (2)
N12	0.0152 (3)	0.0104 (3)	0.0173 (3)	−0.0060 (2)	−0.0045 (3)	−0.0010 (2)
C13	0.0133 (3)	0.0105 (3)	0.0157 (3)	−0.0060 (3)	−0.0034 (3)	−0.0010 (3)
C14	0.0131 (3)	0.0095 (3)	0.0133 (3)	−0.0052 (3)	−0.0024 (3)	−0.0009 (2)
C15	0.0163 (4)	0.0106 (3)	0.0148 (3)	−0.0069 (3)	−0.0032 (3)	−0.0016 (3)
C16	0.0154 (4)	0.0090 (3)	0.0234 (4)	−0.0042 (3)	−0.0055 (3)	−0.0005 (3)
C17	0.0198 (4)	0.0136 (4)	0.0231 (4)	−0.0084 (3)	−0.0093 (3)	0.0043 (3)
C18	0.0217 (5)	0.0328 (6)	0.0248 (5)	−0.0124 (4)	−0.0053 (4)	0.0068 (4)
C19	0.0343 (6)	0.0158 (4)	0.0408 (6)	−0.0114 (4)	−0.0217 (5)	0.0108 (4)
C21	0.0147 (3)	0.0138 (3)	0.0130 (3)	−0.0066 (3)	−0.0032 (3)	−0.0005 (3)
C22	0.0192 (4)	0.0156 (4)	0.0173 (4)	−0.0065 (3)	−0.0055 (3)	−0.0001 (3)
C23	0.0205 (4)	0.0193 (4)	0.0167 (4)	−0.0052 (3)	−0.0062 (3)	0.0019 (3)
C24	0.0184 (4)	0.0256 (5)	0.0136 (3)	−0.0086 (3)	−0.0057 (3)	0.0014 (3)
C25	0.0271 (5)	0.0221 (5)	0.0214 (4)	−0.0105 (4)	−0.0115 (4)	−0.0012 (4)
C26	0.0253 (5)	0.0174 (4)	0.0227 (4)	−0.0085 (3)	−0.0113 (4)	−0.0019 (3)
O2	0.0232 (3)	0.0154 (3)	0.0200 (3)	−0.0106 (3)	0.0036 (3)	−0.0009 (2)
N1	0.0186 (4)	0.0151 (3)	0.0189 (3)	−0.0112 (3)	0.0038 (3)	−0.0040 (3)
N2	0.0245 (4)	0.0158 (4)	0.0248 (4)	−0.0058 (3)	0.0029 (3)	−0.0043 (3)
O1'	0.0177 (3)	0.0098 (2)	0.0138 (3)	−0.0062 (2)	−0.0004 (2)	−0.0008 (2)
C2'	0.0142 (3)	0.0107 (3)	0.0121 (3)	−0.0059 (3)	−0.0024 (3)	−0.0012 (3)
C3'	0.0124 (3)	0.0100 (3)	0.0145 (3)	−0.0054 (3)	−0.0019 (3)	−0.0005 (3)
C4'	0.0133 (3)	0.0096 (3)	0.0135 (3)	−0.0051 (3)	−0.0022 (3)	−0.0011 (3)
C4A'	0.0147 (3)	0.0105 (3)	0.0120 (3)	−0.0057 (3)	−0.0027 (3)	−0.0008 (2)
C5'	0.0157 (4)	0.0138 (4)	0.0150 (3)	−0.0076 (3)	−0.0012 (3)	−0.0023 (3)
C6'	0.0138 (4)	0.0162 (4)	0.0182 (4)	−0.0070 (3)	−0.0002 (3)	−0.0046 (3)
C7'	0.0152 (4)	0.0124 (3)	0.0145 (3)	−0.0048 (3)	−0.0028 (3)	−0.0032 (3)
C8'	0.0202 (4)	0.0098 (3)	0.0145 (3)	−0.0045 (3)	−0.0032 (3)	−0.0020 (3)
C8A'	0.0144 (3)	0.0107 (3)	0.0125 (3)	−0.0058 (3)	−0.0030 (3)	−0.0008 (3)
C9'	0.0155 (4)	0.0123 (3)	0.0154 (3)	−0.0064 (3)	−0.0025 (3)	0.0006 (3)
C10'	0.0206 (4)	0.0158 (4)	0.0233 (4)	−0.0037 (3)	−0.0003 (3)	−0.0074 (3)
C11'	0.0177 (4)	0.0227 (4)	0.0162 (4)	−0.0067 (3)	−0.0058 (3)	−0.0030 (3)
N11'	0.0148 (3)	0.0097 (3)	0.0149 (3)	−0.0059 (2)	−0.0043 (2)	−0.0004 (2)
N12'	0.0144 (3)	0.0102 (3)	0.0171 (3)	−0.0055 (2)	−0.0041 (2)	−0.0003 (2)
C13'	0.0133 (3)	0.0102 (3)	0.0161 (3)	−0.0051 (3)	−0.0025 (3)	−0.0016 (3)
C14'	0.0124 (3)	0.0104 (3)	0.0139 (3)	−0.0057 (3)	−0.0017 (3)	−0.0014 (3)
C15'	0.0154 (4)	0.0108 (3)	0.0156 (3)	−0.0062 (3)	−0.0038 (3)	−0.0013 (3)
C16'	0.0152 (4)	0.0106 (3)	0.0190 (4)	−0.0046 (3)	−0.0032 (3)	−0.0014 (3)
C17'	0.0149 (4)	0.0119 (3)	0.0190 (4)	−0.0058 (3)	−0.0049 (3)	0.0010 (3)
C18'	0.0157 (4)	0.0247 (5)	0.0235 (4)	−0.0065 (4)	−0.0029 (3)	0.0027 (4)
C19'	0.0216 (4)	0.0146 (4)	0.0267 (5)	−0.0067 (3)	−0.0090 (4)	0.0043 (3)
C21'	0.0137 (3)	0.0142 (3)	0.0136 (3)	−0.0051 (3)	−0.0029 (3)	−0.0021 (3)
C22'	0.0239 (4)	0.0185 (4)	0.0154 (4)	−0.0108 (3)	−0.0056 (3)	0.0024 (3)
C23'	0.0266 (5)	0.0225 (4)	0.0157 (4)	−0.0097 (4)	−0.0083 (3)	0.0039 (3)
C24'	0.0209 (4)	0.0249 (5)	0.0149 (4)	−0.0071 (4)	−0.0065 (3)	−0.0010 (3)
C25'	0.0171 (4)	0.0212 (4)	0.0211 (4)	−0.0066 (3)	−0.0070 (3)	−0.0038 (3)
C26'	0.0172 (4)	0.0153 (4)	0.0214 (4)	−0.0070 (3)	−0.0064 (3)	0.0002 (3)
O2'	0.0289 (4)	0.0157 (3)	0.0212 (3)	−0.0114 (3)	0.0048 (3)	−0.0012 (3)
N1'	0.0194 (4)	0.0146 (3)	0.0170 (3)	−0.0106 (3)	0.0009 (3)	−0.0026 (3)
N2'	0.0231 (4)	0.0136 (3)	0.0246 (4)	−0.0049 (3)	0.0006 (3)	−0.0019 (3)

### 2-Amino-4-(3-isobutyl-1-phenyl-1H-pyrazol-4-yl)-7,7-dimethyl-5-oxo-5,6,7,8-tetrahydro-4H-chromene-3-carbonitrile Geometric parameters (Å, º)

**Table:**

O1—C2	1.3663 (11)	O1'—C2'	1.3672 (11)
O1—C8A	1.3760 (11)	O1'—C8A'	1.3736 (11)
C2—N1	1.3474 (12)	C2'—N1'	1.3505 (12)
C2—C3	1.3623 (12)	C2'—C3'	1.3629 (12)
C3—C9	1.4125 (12)	C3'—C9'	1.4158 (12)
C3—C4	1.5207 (12)	C3'—C4'	1.5206 (12)
C4—C4A	1.5085 (12)	C4'—C4A'	1.5064 (12)
C4—C14	1.5146 (12)	C4'—C14'	1.5129 (12)
C4—H4	1.0000	C4'—H4'	1.0000
C4A—C8A	1.3404 (12)	C4A'—C8A'	1.3446 (12)
C4A—C5	1.4753 (12)	C4A'—C5'	1.4744 (13)
C5—O2	1.2271 (11)	C5'—O2'	1.2244 (12)
C5—C6	1.5073 (13)	C5'—C6'	1.5086 (13)
C6—C7	1.5375 (13)	C6'—C7'	1.5388 (13)
C6—H6A	0.9900	C6'—H6'A	0.9900
C6—H6B	0.9900	C6'—H6'B	0.9900
C7—C10	1.5289 (13)	C7'—C10'	1.5287 (13)
C7—C11	1.5346 (13)	C7'—C11'	1.5349 (13)
C7—C8	1.5409 (13)	C7'—C8'	1.5401 (13)
C8—C8A	1.4924 (12)	C8'—C8A'	1.4936 (12)
C8—H8A	0.9900	C8'—H8'A	0.9900
C8—H8B	0.9900	C8'—H8'B	0.9900
C9—N2	1.1603 (13)	C9'—N2'	1.1610 (13)
C10—H10A	0.9800	C10'—H10D	0.9800
C10—H10B	0.9800	C10'—H10E	0.9800
C10—H10C	0.9800	C10'—H10F	0.9800
C11—H11A	0.9800	C11'—H11D	0.9800
C11—H11B	0.9800	C11'—H11E	0.9800
C11—H11C	0.9800	C11'—H11F	0.9800
N11—C15	1.3644 (12)	N11'—C15'	1.3627 (12)
N11—N12	1.3644 (11)	N11'—N12'	1.3631 (11)
N11—C21	1.4201 (12)	N11'—C21'	1.4206 (12)
N12—C13	1.3368 (11)	N12'—C13'	1.3371 (11)
C13—C14	1.4176 (12)	C13'—C14'	1.4183 (12)
C13—C16	1.4954 (12)	C13'—C16'	1.4999 (12)
C14—C15	1.3800 (12)	C14'—C15'	1.3809 (12)
C15—H15	0.9500	C15'—H15'	0.9500
C16—C17	1.5420 (14)	C16'—C17'	1.5408 (13)
C16—H16A	0.9900	C16'—H16C	0.9900
C16—H16B	0.9900	C16'—H16D	0.9900
C17—C19	1.5245 (14)	C17'—C19'	1.5229 (13)
C17—C18	1.5250 (15)	C17'—C18'	1.5242 (14)
C17—H17	1.0000	C17'—H17'	1.0000
C18—H18A	0.9800	C18'—H18D	0.9800
C18—H18B	0.9800	C18'—H18E	0.9800
C18—H18C	0.9800	C18'—H18F	0.9800
C19—H19A	0.9800	C19'—H19D	0.9800
C19—H19B	0.9800	C19'—H19E	0.9800
C19—H19C	0.9800	C19'—H19F	0.9800
C21—C26	1.3921 (13)	C21'—C22'	1.3943 (13)
C21—C22	1.3953 (13)	C21'—C26'	1.3971 (13)
C22—C23	1.3927 (13)	C22'—C23'	1.3900 (14)
C22—H22	0.9500	C22'—H22'	0.9500
C23—C24	1.3897 (15)	C23'—C24'	1.3878 (15)
C23—H23	0.9500	C23'—H23'	0.9500
C24—C25	1.3860 (15)	C24'—C25'	1.3897 (15)
C24—H24	0.9500	C24'—H24'	0.9500
C25—C26	1.3940 (14)	C25'—C26'	1.3955 (13)
C25—H25	0.9500	C25'—H25'	0.9500
C26—H26	0.9500	C26'—H26'	0.9500
N1—H01	0.868 (16)	N1'—H01'	0.878 (17)
N1—H02	0.886 (18)	N1'—H02'	0.899 (18)
			
C2—O1—C8A	118.60 (7)	C2'—O1'—C8A'	118.45 (7)
N1—C2—C3	127.39 (8)	N1'—C2'—C3'	127.81 (8)
N1—C2—O1	110.45 (7)	N1'—C2'—O1'	110.10 (8)
C3—C2—O1	122.16 (8)	C3'—C2'—O1'	122.08 (8)
C2—C3—C9	118.87 (8)	C2'—C3'—C9'	119.24 (8)
C2—C3—C4	122.98 (8)	C2'—C3'—C4'	122.79 (8)
C9—C3—C4	118.07 (7)	C9'—C3'—C4'	117.79 (7)
C4A—C4—C14	111.88 (7)	C4A'—C4'—C14'	111.68 (7)
C4A—C4—C3	108.28 (7)	C4A'—C4'—C3'	108.14 (7)
C14—C4—C3	112.35 (7)	C14'—C4'—C3'	112.21 (7)
C4A—C4—H4	108.1	C4A'—C4'—H4'	108.2
C14—C4—H4	108.1	C14'—C4'—H4'	108.2
C3—C4—H4	108.1	C3'—C4'—H4'	108.2
C8A—C4A—C5	118.36 (8)	C8A'—C4A'—C5'	118.42 (8)
C8A—C4A—C4	123.37 (8)	C8A'—C4A'—C4'	123.05 (8)
C5—C4A—C4	118.22 (7)	C5'—C4A'—C4'	118.48 (7)
O2—C5—C4A	120.32 (8)	O2'—C5'—C4A'	120.11 (8)
O2—C5—C6	122.23 (8)	O2'—C5'—C6'	122.26 (8)
C4A—C5—C6	117.45 (8)	C4A'—C5'—C6'	117.62 (8)
C5—C6—C7	112.86 (7)	C5'—C6'—C7'	112.80 (7)
C5—C6—H6A	109.0	C5'—C6'—H6'A	109.0
C7—C6—H6A	109.0	C7'—C6'—H6'A	109.0
C5—C6—H6B	109.0	C5'—C6'—H6'B	109.0
C7—C6—H6B	109.0	C7'—C6'—H6'B	109.0
H6A—C6—H6B	107.8	H6'A—C6'—H6'B	107.8
C10—C7—C11	109.22 (7)	C10'—C7'—C11'	109.48 (8)
C10—C7—C6	109.50 (7)	C10'—C7'—C6'	109.23 (8)
C11—C7—C6	109.73 (8)	C11'—C7'—C6'	109.64 (8)
C10—C7—C8	109.60 (8)	C10'—C7'—C8'	109.81 (8)
C11—C7—C8	110.03 (7)	C11'—C7'—C8'	110.00 (8)
C6—C7—C8	108.76 (7)	C6'—C7'—C8'	108.67 (7)
C8A—C8—C7	113.14 (7)	C8A'—C8'—C7'	113.06 (7)
C8A—C8—H8A	109.0	C8A'—C8'—H8'A	109.0
C7—C8—H8A	109.0	C7'—C8'—H8'A	109.0
C8A—C8—H8B	109.0	C8A'—C8'—H8'B	109.0
C7—C8—H8B	109.0	C7'—C8'—H8'B	109.0
H8A—C8—H8B	107.8	H8'A—C8'—H8'B	107.8
C4A—C8A—O1	122.88 (8)	C4A'—C8A'—O1'	122.97 (8)
C4A—C8A—C8	126.01 (8)	C4A'—C8A'—C8'	125.76 (8)
O1—C8A—C8	111.11 (7)	O1'—C8A'—C8'	111.26 (7)
N2—C9—C3	177.46 (11)	N2'—C9'—C3'	177.58 (10)
C7—C10—H10A	109.5	C7'—C10'—H10D	109.5
C7—C10—H10B	109.5	C7'—C10'—H10E	109.5
H10A—C10—H10B	109.5	H10D—C10'—H10E	109.5
C7—C10—H10C	109.5	C7'—C10'—H10F	109.5
H10A—C10—H10C	109.5	H10D—C10'—H10F	109.5
H10B—C10—H10C	109.5	H10E—C10'—H10F	109.5
C7—C11—H11A	109.5	C7'—C11'—H11D	109.5
C7—C11—H11B	109.5	C7'—C11'—H11E	109.5
H11A—C11—H11B	109.5	H11D—C11'—H11E	109.5
C7—C11—H11C	109.5	C7'—C11'—H11F	109.5
H11A—C11—H11C	109.5	H11D—C11'—H11F	109.5
H11B—C11—H11C	109.5	H11E—C11'—H11F	109.5
C15—N11—N12	111.27 (7)	C15'—N11'—N12'	111.41 (7)
C15—N11—C21	128.02 (8)	C15'—N11'—C21'	127.54 (8)
N12—N11—C21	120.68 (7)	N12'—N11'—C21'	121.01 (7)
C13—N12—N11	105.58 (7)	C13'—N12'—N11'	105.55 (7)
N12—C13—C14	110.94 (8)	N12'—C13'—C14'	110.91 (8)
N12—C13—C16	120.35 (8)	N12'—C13'—C16'	119.96 (8)
C14—C13—C16	128.71 (8)	C14'—C13'—C16'	129.11 (8)
C15—C14—C13	104.92 (7)	C15'—C14'—C13'	104.89 (8)
C15—C14—C4	127.10 (8)	C15'—C14'—C4'	126.23 (8)
C13—C14—C4	127.91 (8)	C13'—C14'—C4'	128.86 (8)
N11—C15—C14	107.28 (8)	N11'—C15'—C14'	107.23 (8)
N11—C15—H15	126.4	N11'—C15'—H15'	126.4
C14—C15—H15	126.4	C14'—C15'—H15'	126.4
C13—C16—C17	115.49 (8)	C13'—C16'—C17'	115.82 (7)
C13—C16—H16A	108.4	C13'—C16'—H16C	108.3
C17—C16—H16A	108.4	C17'—C16'—H16C	108.3
C13—C16—H16B	108.4	C13'—C16'—H16D	108.3
C17—C16—H16B	108.4	C17'—C16'—H16D	108.3
H16A—C16—H16B	107.5	H16C—C16'—H16D	107.4
C19—C17—C18	110.65 (9)	C19'—C17'—C18'	110.31 (8)
C19—C17—C16	109.24 (9)	C19'—C17'—C16'	109.42 (8)
C18—C17—C16	111.99 (8)	C18'—C17'—C16'	111.66 (8)
C19—C17—H17	108.3	C19'—C17'—H17'	108.5
C18—C17—H17	108.3	C18'—C17'—H17'	108.5
C16—C17—H17	108.3	C16'—C17'—H17'	108.5
C17—C18—H18A	109.5	C17'—C18'—H18D	109.5
C17—C18—H18B	109.5	C17'—C18'—H18E	109.5
H18A—C18—H18B	109.5	H18D—C18'—H18E	109.5
C17—C18—H18C	109.5	C17'—C18'—H18F	109.5
H18A—C18—H18C	109.5	H18D—C18'—H18F	109.5
H18B—C18—H18C	109.5	H18E—C18'—H18F	109.5
C17—C19—H19A	109.5	C17'—C19'—H19D	109.5
C17—C19—H19B	109.5	C17'—C19'—H19E	109.5
H19A—C19—H19B	109.5	H19D—C19'—H19E	109.5
C17—C19—H19C	109.5	C17'—C19'—H19F	109.5
H19A—C19—H19C	109.5	H19D—C19'—H19F	109.5
H19B—C19—H19C	109.5	H19E—C19'—H19F	109.5
C26—C21—C22	120.11 (8)	C22'—C21'—C26'	120.39 (8)
C26—C21—N11	119.82 (8)	C22'—C21'—N11'	120.02 (8)
C22—C21—N11	120.06 (8)	C26'—C21'—N11'	119.59 (8)
C23—C22—C21	119.44 (9)	C23'—C22'—C21'	119.29 (9)
C23—C22—H22	120.3	C23'—C22'—H22'	120.4
C21—C22—H22	120.3	C21'—C22'—H22'	120.4
C24—C23—C22	120.64 (9)	C24'—C23'—C22'	121.06 (10)
C24—C23—H23	119.7	C24'—C23'—H23'	119.5
C22—C23—H23	119.7	C22'—C23'—H23'	119.5
C25—C24—C23	119.60 (9)	C23'—C24'—C25'	119.27 (9)
C25—C24—H24	120.2	C23'—C24'—H24'	120.4
C23—C24—H24	120.2	C25'—C24'—H24'	120.4
C24—C25—C26	120.43 (10)	C24'—C25'—C26'	120.72 (9)
C24—C25—H25	119.8	C24'—C25'—H25'	119.6
C26—C25—H25	119.8	C26'—C25'—H25'	119.6
C21—C26—C25	119.76 (9)	C25'—C26'—C21'	119.24 (9)
C21—C26—H26	120.1	C25'—C26'—H26'	120.4
C25—C26—H26	120.1	C21'—C26'—H26'	120.4
C2—N1—H01	121.3 (10)	C2'—N1'—H01'	119.6 (11)
C2—N1—H02	120.3 (12)	C2'—N1'—H02'	118.3 (12)
H01—N1—H02	116.3 (15)	H01'—N1'—H02'	114.0 (15)
			
C8A—O1—C2—N1	−172.40 (8)	C8A'—O1'—C2'—N1'	−172.34 (8)
C8A—O1—C2—C3	8.30 (12)	C8A'—O1'—C2'—C3'	8.38 (12)
N1—C2—C3—C9	−0.17 (14)	N1'—C2'—C3'—C9'	0.87 (14)
O1—C2—C3—C9	179.01 (8)	O1'—C2'—C3'—C9'	−179.98 (8)
N1—C2—C3—C4	−177.01 (9)	N1'—C2'—C3'—C4'	−174.29 (9)
O1—C2—C3—C4	2.16 (13)	O1'—C2'—C3'—C4'	4.85 (13)
C2—C3—C4—C4A	−11.67 (11)	C2'—C3'—C4'—C4A'	−15.29 (11)
C9—C3—C4—C4A	171.46 (8)	C9'—C3'—C4'—C4A'	169.48 (8)
C2—C3—C4—C14	112.40 (9)	C2'—C3'—C4'—C14'	108.34 (9)
C9—C3—C4—C14	−64.47 (10)	C9'—C3'—C4'—C14'	−66.90 (10)
C14—C4—C4A—C8A	−111.84 (9)	C14'—C4'—C4A'—C8A'	−109.20 (10)
C3—C4—C4A—C8A	12.51 (11)	C3'—C4'—C4A'—C8A'	14.75 (11)
C14—C4—C4A—C5	70.80 (10)	C14'—C4'—C4A'—C5'	73.12 (10)
C3—C4—C4A—C5	−164.85 (7)	C3'—C4'—C4A'—C5'	−162.94 (8)
C8A—C4A—C5—O2	−165.26 (9)	C8A'—C4A'—C5'—O2'	−166.71 (9)
C4—C4A—C5—O2	12.24 (13)	C4'—C4A'—C5'—O2'	11.09 (13)
C8A—C4A—C5—C6	15.18 (12)	C8A'—C4A'—C5'—C6'	14.24 (12)
C4—C4A—C5—C6	−167.32 (7)	C4'—C4A'—C5'—C6'	−167.97 (8)
O2—C5—C6—C7	139.75 (9)	O2'—C5'—C6'—C7'	141.01 (10)
C4A—C5—C6—C7	−40.70 (11)	C4A'—C5'—C6'—C7'	−39.95 (11)
C5—C6—C7—C10	173.93 (8)	C5'—C6'—C7'—C10'	174.16 (8)
C5—C6—C7—C11	−66.21 (10)	C5'—C6'—C7'—C11'	−65.89 (10)
C5—C6—C7—C8	54.19 (10)	C5'—C6'—C7'—C8'	54.37 (10)
C10—C7—C8—C8A	−163.90 (8)	C10'—C7'—C8'—C8A'	−164.55 (8)
C11—C7—C8—C8A	75.99 (10)	C11'—C7'—C8'—C8A'	74.90 (10)
C6—C7—C8—C8A	−44.22 (10)	C6'—C7'—C8'—C8A'	−45.13 (10)
C5—C4A—C8A—O1	173.49 (8)	C5'—C4A'—C8A'—O1'	173.95 (8)
C4—C4A—C8A—O1	−3.87 (13)	C4'—C4A'—C8A'—O1'	−3.73 (13)
C5—C4A—C8A—C8	−5.90 (13)	C5'—C4A'—C8A'—C8'	−5.65 (13)
C4—C4A—C8A—C8	176.74 (8)	C4'—C4A'—C8A'—C8'	176.66 (8)
C2—O1—C8A—C4A	−7.55 (12)	C2'—O1'—C8A'—C4A'	−9.07 (12)
C2—O1—C8A—C8	171.92 (7)	C2'—O1'—C8A'—C8'	170.59 (7)
C7—C8—C8A—C4A	21.95 (13)	C7'—C8'—C8A'—C4A'	22.65 (13)
C7—C8—C8A—O1	−157.51 (7)	C7'—C8'—C8A'—O1'	−156.99 (7)
C15—N11—N12—C13	0.42 (10)	C15'—N11'—N12'—C13'	0.31 (10)
C21—N11—N12—C13	178.71 (8)	C21'—N11'—N12'—C13'	178.48 (8)
N11—N12—C13—C14	0.19 (10)	N11'—N12'—C13'—C14'	0.26 (10)
N11—N12—C13—C16	−179.64 (8)	N11'—N12'—C13'—C16'	−178.52 (8)
N12—C13—C14—C15	−0.71 (10)	N12'—C13'—C14'—C15'	−0.70 (10)
C16—C13—C14—C15	179.11 (9)	C16'—C13'—C14'—C15'	177.93 (9)
N12—C13—C14—C4	−177.87 (8)	N12'—C13'—C14'—C4'	−178.74 (8)
C16—C13—C14—C4	1.95 (15)	C16'—C13'—C14'—C4'	−0.11 (15)
C4A—C4—C14—C15	49.07 (12)	C4A'—C4'—C14'—C15'	53.24 (12)
C3—C4—C14—C15	−72.98 (11)	C3'—C4'—C14'—C15'	−68.38 (11)
C4A—C4—C14—C13	−134.37 (9)	C4A'—C4'—C14'—C13'	−129.11 (9)
C3—C4—C14—C13	103.58 (10)	C3'—C4'—C14'—C13'	109.27 (10)
N12—N11—C15—C14	−0.87 (10)	N12'—N11'—C15'—C14'	−0.75 (10)
C21—N11—C15—C14	−179.01 (9)	C21'—N11'—C15'—C14'	−178.78 (8)
C13—C14—C15—N11	0.92 (10)	C13'—C14'—C15'—N11'	0.85 (10)
C4—C14—C15—N11	178.12 (8)	C4'—C14'—C15'—N11'	178.96 (8)
N12—C13—C16—C17	−106.58 (10)	N12'—C13'—C16'—C17'	−104.43 (10)
C14—C13—C16—C17	73.62 (12)	C14'—C13'—C16'—C17'	77.05 (12)
C13—C16—C17—C19	179.89 (8)	C13'—C16'—C17'—C19'	176.15 (8)
C13—C16—C17—C18	56.94 (11)	C13'—C16'—C17'—C18'	53.73 (11)
C15—N11—C21—C26	163.71 (10)	C15'—N11'—C21'—C22'	13.50 (14)
C15—N11—C21—C22	−15.34 (15)	N12'—N11'—C21'—C22'	−164.36 (9)
N12—N11—C21—C22	166.67 (8)	C15'—N11'—C21'—C26'	−166.43 (9)
N12—N11—C21—C26	−14.27 (13)	N12'—N11'—C21'—C26'	15.71 (13)
C26—C21—C22—C23	1.47 (15)	C26'—C21'—C22'—C23'	−0.70 (15)
N11—C21—C22—C23	−179.48 (9)	N11'—C21'—C22'—C23'	179.37 (9)
C21—C22—C23—C24	−0.53 (15)	C21'—C22'—C23'—C24'	−0.18 (16)
C22—C23—C24—C25	−0.64 (16)	C22'—C23'—C24'—C25'	−0.24 (16)
C23—C24—C25—C26	0.88 (17)	C23'—C24'—C25'—C26'	1.56 (15)
C22—C21—C26—C25	−1.23 (16)	C24'—C25'—C26'—C21'	−2.42 (15)
N11—C21—C26—C25	179.71 (10)	C22'—C21'—C26'—C25'	1.99 (14)
C24—C25—C26—C21	0.05 (17)	N11'—C21'—C26'—C25'	−178.09 (9)

### 2-Amino-4-(3-isobutyl-1-phenyl-1H-pyrazol-4-yl)-7,7-dimethyl-5-oxo-5,6,7,8-tetrahydro-4H-chromene-3-carbonitrile Hydrogen-bond geometry (Å, º)

**Table:**

*D*—H···*A*	*D*—H	H···*A*	*D*···*A*	*D*—H···*A*
N1′—H01′···N12	0.878 (17)	2.115 (17)	2.9895 (11)	173.5 (14)
N1—H01···N12′^i^	0.868 (16)	2.145 (16)	3.0071 (11)	172.0 (15)
C15—H15···N2′^ii^	0.95	2.41	3.3622 (13)	176
C15′—H15′···N2^iii^	0.95	2.43	3.3816 (13)	180
C11—H11*A*···O2′^iv^	0.98	2.47	3.3965 (12)	157
C11′—H11*D*···O2^iv^	0.98	2.59	3.5046 (13)	155

Symmetry codes: (i) *x*+1, *y*+1, *z*; (ii) *x*, *y*+1, *z*; (iii) *x*−1, *y*, *z*; (iv) −*x*+1, −*y*+1, −*z*+2.
